# A Machine Learning Assisted Non-Enzymatic Electrochemical Biosensor to Detect Urea Based on Multi-Walled Carbon Nanotube Functionalized with Copper Oxide Micro-Flowers

**DOI:** 10.3390/bios14100504

**Published:** 2024-10-15

**Authors:** Jitendra B. Zalke, Manish L. Bhaiyya, Pooja A. Jain, Devashree N. Sakharkar, Jayu Kalambe, Nitin P. Narkhede, Mangesh B. Thakre, Dinesh R. Rotake, Madhusudan B. Kulkarni, Shiv Govind Singh

**Affiliations:** 1 Department of Electronics Engineering, Ramdeobaba University, Nagpur 440013, MH, India; zalkej@rknec.edu (J.B.Z.); bhaiyyaml_1@rknec.edu (M.L.B.); kalambej@rknec.edu (J.K.); narkheden@rknec.edu (N.P.N.); 2 Department of Biomedical Engineering, Shri Ramdeobaba College of Engineering and Management, Nagpur 440013, MH, India; jainpa_3@rknec.edu (P.A.J.); sakharkardn@rknec.edu (D.N.S.); 3 Department of Chemistry, D.R.B. Sindhu Mahavidhyalaya, Nagpur 440017, MH, India; mangeshthakre@gmail.com; 4 Department of Electrical Engineering, Indian Institute of Technology, Hyderabad 502284, TG, India; sgsingh@ee.iith.ac.in; 5 Department of Medical Physics, University of Wisconsin-Madison, Madison, WI 53705, USA; 6 Department of Electronics and Communication Engineering, Manipal Institute of Technology, Manipal Academy of Higher Education (MAHE), Manipal 576104, KA, India

**Keywords:** non-enzymatic, urea, copper oxide (CuO) micro-flowers (MFs), machine learning (ML), screen-printed electrodes (SPEs), biomarker, electrochemical detection

## Abstract

Detecting urea is crucial for diagnosing related health conditions and ensuring timely medical intervention. The addition of machine learning (ML) technologies has completely changed the field of biochemical sensing, providing enhanced accuracy and reliability. In the present work, an ML-assisted screen-printed, flexible, electrochemical, non-enzymatic biosensor was proposed to quantify urea concentrations. For the detection of urea, the biosensor was modified with a multi-walled carbon nanotube-zinc oxide (MWCNT-ZnO) nanocomposite functionalized with copper oxide (CuO) micro-flowers (MFs). Further, the CuO-MFs were synthesized using a standard sol-gel approach, and the obtained particles were subjected to various characterization techniques, including X-ray diffraction (XRD), field emission scanning electron microscopy (FESEM), and Fourier transform infrared (FTIR) spectroscopy. The sensor’s performance for urea detection was evaluated by assessing the dependence of peak currents on analyte concentration using cyclic voltammetry (CV) at different scan rates of 50, 75, and 100 mV/s. The designed non-enzymatic biosensor showed an acceptable linear range of operation of 0.5–8 mM, and the limit of detection (LoD) observed was 78.479 nM, which is well aligned with the urea concentration found in human blood and exhibits a good sensitivity of 117.98 mA mM^−1^ cm^−2^. Additionally, different regression-based ML models were applied to determine CV parameters to predict urea concentrations experimentally. ML significantly improves the accuracy and reliability of screen-printed biosensors, enabling accurate predictions of urea levels. Finally, the combination of ML and biosensor design emphasizes not only the high sensitivity and accuracy of the sensor but also its potential for complex non-enzymatic urea detection applications. Future advancements in accurate biochemical sensing technologies are made possible by this strong and dependable methodology.

## 1. Introduction

Urea is an important biomarker in medical diagnostics as it plays a vital role in body detoxifying. It is important for reducing the harmful effects of increased nitrogen levels by altering toxic ammonium ions into urea, which the kidneys then safely excrete through urine [1,2,3]. Hence, urea concentration monitoring is essential for the diagnosis of liver and kidney disorders. Severe conditions such as hyperuricemia, renal abnormalities, acute renal injury, chronic renal disease, nutritional inadequacies, and heart failure can all be indicated by elevated urea levels. Normal blood urea levels range from 1.67 to 7.5 mM, while 342 ± 67 mM is the usual level in 490–2690 mL of urine [4,5,6,7]. Thus, reliable and precise urea detection techniques are essential for clinical diagnosis as well as for preserving overall wellness.

In continuation, the accurate detection of urea ranges beyond medical diagnostics to various fields, which include food safety, agriculture, and cosmetics. Large-scale urea synthesis has changed the manufacture of nitrogen-based fertilizers in agriculture and has had a substantial environmental impact [8,9,10,11]. On the other hand, in the food industry, keeping an eye on urea levels is essential for ensuring food safety, especially with dairy products. Sometimes, urea is added to diluted milk to keep it viscous; this needs to be well-monitored to avoid adulteration [12,13]. A range of well-established analytical methods, such as infrared spectroscopy [14], high-performance liquid chromatography (HPLC) [15], nuclear magnetic resonance (NMR) [6], calorimetry [16], fluorimetry [17], and electrochemiluminescence [18,19,20,21,22], have been used to evaluate urea in real blood samples. Although these techniques yield precise results, they have limitations, including lengthy analysis times, expensive equipment, the requirement for trained operators, and labor-intensive specimen preparation. Blood urea nitrogen analysis is the most common technique for measuring blood urea levels to evaluate azotemia [23,24]. It is frequently carried out in conjunction with serum creatinine assays. However, because of their complexity and resource needs, these traditional methods are not always feasible for quick on-site testing [25].

Alternately, electrochemical sensing approaches provide simpler, more cost-effective, and efficient methods to detect urea in blood. These techniques can be used in the food industry, medical field, military, and study of plant biology, among other fields [26,27,28,29,30,31,32]. Recently, screen-printed electrode (SPE)-based electrochemical biosensors have gained attention for their potential in rapid, sensitive, portable, cost-effective, and precise investigations. Moreover, screen-printing has been suggested as a mass-producible, inexpensive, dependable, single-use sensor technique for on-site monitoring for the past thirty years. SPEs allow the coupling of many carbon-based electrodes with functionalized compounds in an inexpensive, repeatable, and disposable arrangement. When combined with SPEs, electrochemical biosensors can provide a practical substitute for conventional analytical methods for in-field screening and monitoring. Generally, SPEs consist of an electrochemical cell printed on a solid substrate with three electrodes: the reference electrode (RE), counter electrode (CE), and working electrode (WE). These biosensors fall under the following categories: impedimetric, voltammetry, amperometric, and potentiometric [27,33]. When using enzymatic or non-enzymatic techniques for electrochemical detection, urea is found by monitoring redox reactions [23,24,34,35].

The selection of working electrode materials is a critical step in the development of efficient electrochemical sensing platforms. This choice directly affects sensor performance attributes such as low cost, high electrocatalysis, sensitivity, selectivity, stability, electrical conductivity, and biocompatibility. Numerous nanostructured materials, including nanopores, nanoparticles, nanofibers, nanowires, and nanotubes, have been thoroughly studied by researchers [36,37,38]. Carbon-based materials, including graphene, reduced graphene oxide, and carbon nanotubes, as well as metal oxide nanostructures such as zinc oxide, nickel oxide, manganese dioxide, and copper oxide, have attracted considerable interest because of their distinct electrochemical, catalytic, and electrical characteristics. These materials can be accurately altered in terms of their physical structure and surface properties, which is crucial for improving the performance of sensors. Graphene and its derivatives, renowned for their exceptional electrical conductivity and expansive surface area, are especially well-suited for sensors that necessitate fast electron transmission. Moreover, metal oxide nanostructures provide customized chemical reactivity and stability, which are crucial for detecting certain analytes. By adjusting the structure and surface characteristics of these materials, it becomes possible to tailor them according to the unique needs of sensors. This leads to enhancements in sensitivity, selectivity, and overall sensor performance. The capacity to adapt and regulate their properties makes carbon-based and metal oxide materials essential for the development of sensor technologies in environmental monitoring, biomedical diagnostics, and industrial process control applications [39,40,41,42,43]. Upon combining them with electrochemical electrodes and with enhanced conductivity, catalytic activity, and binding affinity for target biomolecules [43], these characteristics enhance the detection signal and make it easier for analytes to react on electrodes enhanced with metallic nanoparticles. As an alternative, attaching ZnO NPs to the MWCNT surface causes the network to form nanotube (NT) and nanoparticle (NP) combinations, which substantially varies the electrical conductivity [32].

In enzymatic urea sensors, the urease enzyme combined with various metal oxide nanocomposite materials has shown potential for urea detection; however, practical applications are limited by issues such as weak conductivity, a narrow detection range, and high urea detection thresholds. These difficulties highlight the continuous attempts to increase metal oxide nanostructures’ effectiveness and expand their range of applications in sensing technologies. Several innovative biosensors for urea detection have been developed using various advanced techniques and materials [44,45,46,47,48]. Enzymatic urea sensors encounter challenges such as enzyme immobilization difficulties, high costs, reproducibility issues, and limitations in operational parameters such as temperature, pH, and humidity [49]. Consequently, non-enzymatic electrochemical biosensors have been explored for urea detection. In this approach, urea undergoes oxidation/reduction on suitable electrodes [50,51]. Researchers innovatively developed an electrochemical sensor using a composite of MWCNT, SWCNT, graphene, and polyaniline (PANi) without enzymatic involvement. The synthesis involved grafting PANi onto graphene through CV, which was validated using Raman spectroscopy. This sensor exhibited enhanced sensitivity and a reduced detection limit, as well as demonstrated outstanding reproducibility, specificity, and durability. It effectively quantified urea levels in both water and milk samples [49]. This advancement offers a straightforward and cost-effective approach applicable to clinical diagnostics, milk quality assessment, pesticide production, and environmental monitoring for pollutants.

A new biosensor for urea detection was created using a porous composite catalyst composed of nickel-metal organic Framework (Ni–MOF) and MWCNTs. The electrode, fabricated on ITO glass, exhibited strong performance in detecting urea, boasting a high sensitivity of 685 μAmM^−1^ cm^−2^ and a rapid response time of just 10 s. The biosensor achieved a LoD of 3 μM and demonstrated stability over a storage period of 30 days. The combination of Ni–MOF and MWCNTs in the electrode design leverages their synergistic effects, significantly enhancing the electrocatalytic activity for both urea oxidation and reduction reactions [52]. Similarly, a glossy carbon electrode (GCE) incorporating silver-doped single-walled carbon nanotubes (SWCNTs) was developed using a simplified thermal reduction process. This electrode exhibited a linear range from 66.0 nM to 20.6 mM for urea detection, with a sensitivity of 141.0 μAmM^−1^ cm^−1^ and a LoD of 4.70 nM. The electrode’s performance was evaluated in practical scenarios, successfully measuring urea levels in tap water and dairy milk [53]. These advancements highlight the potential of composite catalysts and nanomaterials in developing efficient biosensing platforms for urea detection, with implications for various applications, including environmental monitoring and food quality assessment.

An ultrathin Ni-MOF nanobelt sensor showed superior efficiency with a linear range of 0.01–7.0 mM with LoD of 2.23 μM and sensitivity of 118.77 μA mM^−1^ cm^−2^ for urea in biological and environmental samples [54]. A GCE modified with nickel cobalt oxide (NiCo_2_O_4_) nanoneedles, synthesized via a low-temperature aqueous method, was developed for non-enzymatic urea detection. This sensor offers a linear response R^2^ = 0.99 over 0.01–5 mM and a LoD of 1.0 µM. It overcomes NiO and Co_3_O_4_ nanoparticles’ poor conductivity, providing a cost-effective, highly selective urea estimation tool [55]. An Ag/NiOOH nanorods-modified electrode was developed for non-enzymatic urea detection, operating effectively in neutral pH. It shows a higher sensitivity of 233.7 μAmM^−1^ cm^−2^ over a linear range of 0.2–26.0 mM, with a quick response time of ~3.0 s and a LoD of 5.0 μM in neutral phosphate-buffered saline [56].

Table 1 shows the MWCNT-ZnO/CuO-MFs modified non-enzymatic biosensors with earlier reported biosensors for urea detection and various electrode materials used, emphasizing the critical role of nanomaterials in enhancing sensor performance. Sensitivity, limit of detection (LOD), and the linear range are the primary factors influencing the effectiveness of these sensors. Among the materials, Ag-N-SWCNTs exhibit the lowest LOD (4.7 nM) and impressive sensitivity (141 μAmM^−1^ cm^−2^), making them highly effective for detecting even minute concentrations of urea over a wide linear range (66 nM to 20.6 mM). Carbon nanotubes (CNTs), both single-walled (SWCNTs) and multi-walled (MWCNTs), are widely used because of their high electrical conductivity and surface area. For instance, Ni-MOF/MWCNTs show a balance of high sensitivity (685 μAmM^−1^ cm^−2^) and low LOD (3 μM). The use of metal oxides such as NiO and CuO combined with CNTs further improves sensor efficiency. The integration of machine learning (ML) in the MWCNT-ZnO/CuO-MFs electrode underscores the future potential of using ML algorithms for sensor optimization. This sensor achieves high sensitivity (117.98 mA mM^−1^ cm^−2^) and a very low LOD (78.479 nM), highlighting how ML can assist in better calibration and data processing.

The integration of machine learning (ML) and electrochemical sensing is becoming an innovative approach that offers an unmatched ability to decode complex data patterns. Large-scale electrochemical data sets can be evaluated by ML algorithms, which can also identify minor correlations and trends that conventional methods might miss. This results in more accurate measurements that address important urea detection difficulties such as selectivity, specificity, and sensitivity. Moreover, ML has the capacity to adjust and enhance sensor performance over time, guaranteeing reliable and superior results. In addition to improving urea detection, the combination of ML and electrochemical sensing opens new possibilities for quick, precise, and scalable biosensing applications. This novel approach shows promise in a variety of sectors, including clinical diagnostics, environmental monitoring, and industrial process control, transforming how we detect and analyze biological compounds [63,64,65].

This study aims to pioneer the development of an ML-assisted, flexible, electrochemical, non-enzymatic biosensor for precise urea concentration detection. Leveraging a novel MWCNT-ZnO nanocomposite functionalized with CuO-MFs, we seek to enhance sensitivity and operational performance, addressing key challenges faced by traditional biosensors. Our comprehensive evaluation incorporates various characterization techniques and electrochemical CV analysis to validate the biosensor’s effectiveness. Furthermore, by integrating advanced ML models to predict urea concentrations from experimental data, we enhance the sensor’s accuracy and reliability. This research not only demonstrates the transformative potential of ML in sensor technology but also paves the way for innovative applications in clinical diagnostics, environmental monitoring, and food safety. The findings of this study hold significant promise for advancing biochemical sensing technologies, ultimately contributing to improved health outcomes and more effective monitoring of urea levels in diverse settings.

## 2. Materials and Method

### 2.1. Chemicals Material Used

MWCNT-ZnO nanofibers were synthesized at IIT Hyderabad. Potassium ferrocyanide, potassium ferricyanide, ethanol, Nafion, copper oxide, urea, and Whatman grade-1 filter paper were sourced from Sigma-Aldrich, Bommasandra, India. A screen-printed electrode (integrated graphene IG-GII-SENS-01) with a 3D graphene foam WE, Ag/AgCl RE, and 3D graphene foam CE was used. Analytical grade copper nitrate trihydrate and sodium hydroxide (98% purity) were obtained from Sigma-Aldrich and Merck, Mumbai, India. Distilled and deionized water was used.

### 2.2. Methodology

**Figure 1:**
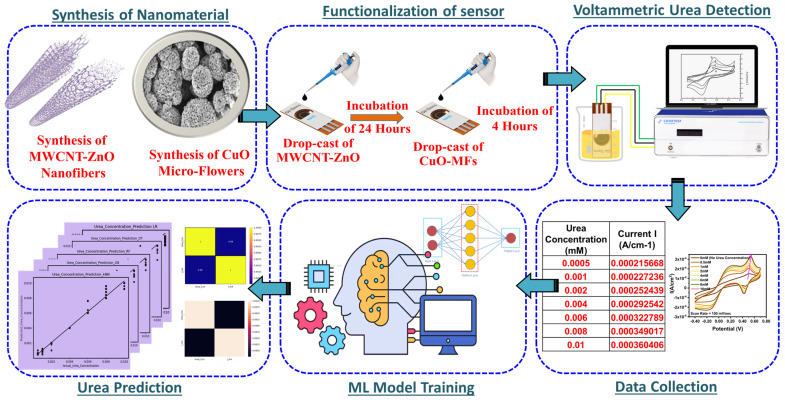
Process steps to detect urea, based on MWCNT-ZnO functionalized with novel CuO-MF and ML-approach.

Figure 1 displays the pictorial representation of the whole course flow and mechanism for detecting urea non-enzymatically, based on MWCNT-ZnO composite nanofibers functionalized with novel copper oxide micro-flowers and a machine learning approach. The various steps involved are a synthesis of MWCNT-ZnO, synthesis of copper oxide micro-flowers, functionalization of screen-printed sensors, test setup for detection of urea using an electrochemical workstation, and ML model for predictive analysis of urea concentration. The successive sections express each process in detail.

#### 2.2.1. Synthesis of MWCNT-ZnO Composite

The composite MWCNT-ZnO nanomaterials were produced using the electrospinning technique. Initially, 30 mg of MWCNTs (5 weight percent relative to ZnO) were ultrasonically dispersed in 10 mL of DMF for 20 min. Then, 0.6 g of polyacrylonitrile (PAN) was added to the MWCNT/DMF solution and ultrasonicated for an additional 5 min. After adding 0.6 g of zinc acetate dihydrate, the liquid was magnetically stirred at 60 °C for four to five hours. For electrospinning, a 5 mL syringe with a 26-gauge metallic needle was filled with this homogenous MWCNT/PAN/ZnO/DMF precursor solution. To produce nanofibers, the electrospinning parameters, 0.7 mL/hr flow rate, and 1.25 KV/cm electric field (15 KV for a distance of 12 cm) were adjusted. In order to prevent the MWCNTs from breaking during the creation of homogenous composite MWCNT-ZnO nanofibers, the nanofiber mats were heated at a rate of 5 °C per minute for two hours at 400 °C in a muffle furnace. The resulting MWCNT-ZnO nanofibers underwent various characterization and morphology studies, which have been detailed in our previous research publications. This method successfully produces robust, uniform composite nanofibers, highlighting the potential for advanced applications in various fields [32].

#### 2.2.2. Preparation of CuO Micro-Flowers

**Figure 2:**
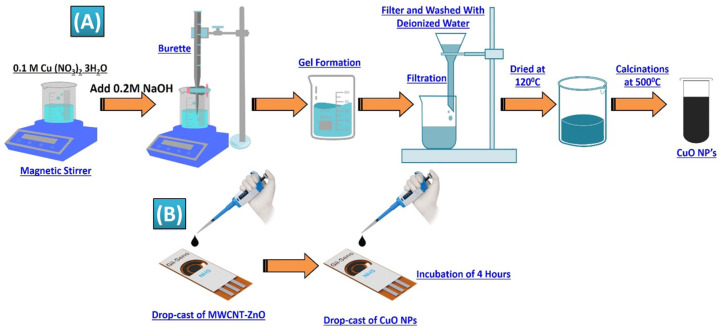
(**A**) Process steps for synthesis and preparation of CuO-MFs. (**B**) Functionalization of Gii-Sens Integrated Graphene SPE.

The sol-gel method was used to create CuO-MF by precipitating copper salt in an alkaline media, utilizing copper nitrate trihydrate (Cu(NO_3_)_2_•3H_2_O) as the precursor. Figure 2A depicts the experimental setup, which consisted of a Corning glass beaker on a magnetic heating plate that was kept at 80 °C. Standard NaOH solution was added dropwise using a 100 mL glass burette that was supported on a platform. The beaker was filled with the copper salt solution that had been made in 100 mL of distilled water. After the solution achieved 80 °C thermal equilibrium, 1 mL of glacial acetic acid was added, and a magnetic stirrer was used to stir the mixture continually. A 0.2 M NaOH solution was added dropwise until the pH hit 11, which denotes an alkaline environment. The synthesis of CuO-MF was verified by the development of a brown-black precipitate. The homogeneous precipitation and micro-flower production was guaranteed by the steady addition of NaOH and continuous stirring. In order to preserve the ideal circumstances for the best possible creation of nanoparticles, the process was closely monitored [66,67]. The steady addition of NaOH and constant stirring ensured uniform precipitation and formation of the nanoparticles. The process was monitored carefully to maintain the desired conditions for optimal nanoparticle formation. This method successfully produced CuO-MFs, which were characterized by their distinctive brown-black color, indicating their successful synthesis of CuO-MFs. The copper oxide precipitate was washed several times with distilled water to remove impurities in the product and then dried in a hot air furnace at 500 °C for 2 h to form a black precipitate. These copper oxide-MFs were investigated by XRD, and morphology was monitored by SEM. Chemical properties were investigated by FTIR analysis [68,69].

#### 2.2.3. Preparation and Functionalization of SPE

Three-electrode Gii-Sens integrated graphene SPE fabricated on flexible polyimide material (commercially available) was used to detect urea. It includes 3D graphene foam as CE and WE and screen-printed Ag/AgCl as RE. The diameter of the WE is 4 mm. Figure 2B illustrates the step-by-step functionalization process of Gii-Sens integrated graphene SPE. First, 5 µL of MWCNT-ZnO composite with a concentration of 2 mg/mL was deposited with a micropipette and allowed to incubate for 24 h at room temperature. A prepared volume of 5 µL CuO-MFs was dropped-cast on the WE and allowed to incubate for nearly four hours at room temperature. The CuO-MFs were prepared by mixing 200 µL of a 2 mM CuO-MFs solution with 200 µL of Nafion and a 10% ethanol solution. This mixture was ultrasonicated for about 30 min to ensure proper dispersion of the CuO-MFs.

#### 2.2.4. Preparation of Urea Stock Solution and Experimental Test Setup

Urea samples with various concentrations ranging from 0.5 to 10 mM were prepared in deionized water. These concentrations were selected to reflect the typical urea levels found in human blood. All electrochemical measurements were performed at room temperature using CorrTest equipment. The three-electrode Gii-Sens integrated graphene SPE, functionalized with MWCNT-ZnO and CuO-MFs, was utilized for cyclic voltammetry (CV) measurements. These measurements were conducted at scan rates of 50, 75, and 100 mV/s, covering a potential range of −0.4 to + 0.7 V. A 5 mM solution of ferroferricyanide (Fe_3_[Fe(CN)_6_]_2_) was employed as the standard redox probe.

## 3. Results and Discussion

### 3.1. Characterization of MWCNT-ZnO Nanofibers

**Figure 3:**
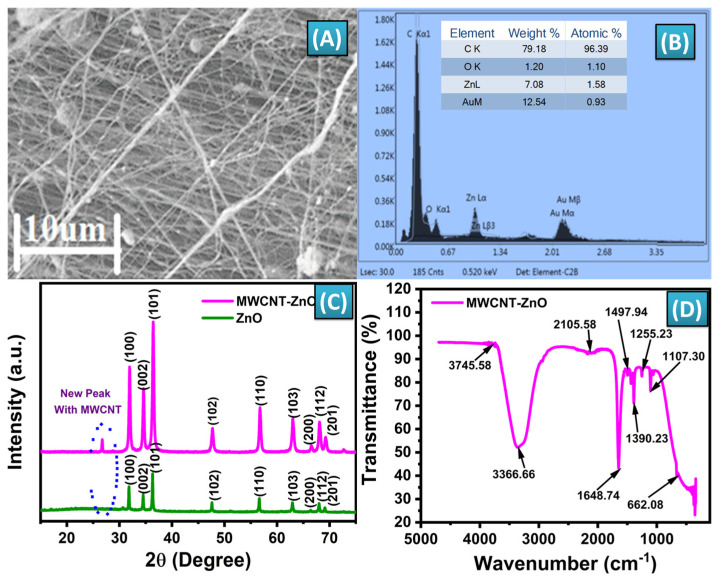
(**A**) SEM image of MWCNT−ZnO showing the exact morphology of nanofibers, (**B**) EDX spectrum of MWCNT−ZnO nanofibers showing the elemental content material composition, (**C**) XRD analysis provides the crystalline structure of the synthesized MWCNT−ZnO nanofibers, (**D**) FTIR analysis showing the functional groups in the MWCNT−ZnO composite.

The MWCNT-ZnO nanofibers were synthesized using the electrospinning technique. The precursor solution consisted of polyacrylonitrile (PAN), N, N-dimethylformamide (DMF), and zinc acetate dihydrate. After fabrication, the nanofibers underwent a high-temperature calcination process at 400 °C to assess their morphology. Figure 3A presents SEM images of the calcined nanofibers, which reveal the formation of MWCNT-ZnO composite nanofibers. This transformation occurs because of the oxidative degradation of PAN and zinc acetate dihydrate during the calcination. Maintaining the calcination temperature at 400 °C was critical to preserve the structural integrity of the carbon nanotubes. The SEM images reveal a relatively uniform distribution of the nanofibers, indicating that the electrospinning process, followed by calcination, produces consistent nanofiber formation. This uniformity is crucial for ensuring predictable performance in sensor applications. The SEM images also indicate that the diameter of the MWCNT-ZnO nanofibers reduced to approximately 180–200 nm. This reduction in size is attributable to the thermal degradation of PAN during the high-temperature process. The degradation not only leads to a decrease in fiber diameter but also increases the surface roughness of the nanofibers, which is a desirable property for various applications. The images demonstrate that the surface of the MWCNT-ZnO nanofibers is rough, which enhances the potential for electron transfer and chemical interactions. This rough surface, attributable to the decomposition of PAN during calcination, is beneficial in applications requiring high surface activity, such as catalysis or sensing. The combination of reduced dimensions and enhanced surface roughness can improve the nanofibers’ performance in applications requiring large surface areas and better interaction with surrounding materials. These properties, evident from the SEM analysis, highlight the potential of MWCNT-ZnO nanofibers for applications requiring enhanced surface area, conductivity, and interaction with various analytes [70].

Energy Dispersive X-ray Spectroscopy (EDX) is a crucial technique used to analyze the elemental composition of MWCNT-ZnO nanofibers synthesized via electrospinning. In this context, EDX identifies the key elements present, such as carbon (C), which originates from the Multi-Walled Carbon Nanotubes (MWCNTs), and zinc (Zn) and oxygen (O), which confirm the formation of ZnO within the nanofibers. As shown in Figure 3B, the spectrum typically shows the weight percentage (wt%) and atomic percentage (at%) of each element, allowing for verification of the expected ratios between ZnO and MWCNTs. For instance, a high carbon content suggests a significant presence of CNTs, while nearly stoichiometric proportions of zinc and oxygen confirm the ZnO phase. Additionally, EDX can provide elemental maps, revealing the spatial distribution of these elements across the nanofibers. In well-synthesized composites, the distribution of zinc and oxygen is expected to be homogeneous, while carbon may be concentrated in areas with more MWCNTs. Moreover, any unexpected elements detected during EDX analysis may indicate contaminants introduced during the electrospinning process. Overall, EDX characterization helps confirm the successful synthesis, composition, and purity of the MWCNT-ZnO nanofibers.

The XRD analysis was performed to assess the crystalline structure of the synthesized MWCNT-ZnO nanofibers, confirming their successful synthesis and structural integrity. As shown in Figure 3C, the distinct peaks observed in the XRD patterns of both ZnO and MWCNT-ZnO nanofibers reflect a well-defined crystalline structure, which is crucial for optimizing their performance in various applications. Figure 3C shows the XRD patterns, with ZnO exhibiting peaks at specific 2θ angles, including 31.5° (100), 34.55° (002), 36.27° (101), 47.6° (102), 56.5° (110), 62.8° (103), 66.29° (200), 68.03° (112), and 69.09° (201). These peaks correspond to the wurtzite crystal structure of ZnO, confirming the formation of the crystalline phase. In addition to these ZnO peaks, the XRD pattern for the MWCNT-ZnO nanofibers shows additional peaks at 27.36° and 45.4°, attributable to the graphite planes C(002) and C(100), respectively. These peaks confirm the presence of MWCNTs within the ZnO nanofiber matrix. The incorporation of MWCNTs into the ZnO nanofibers is thus successfully validated through the distinct XRD peaks of both materials, indicating a coherent and stable composite structure. Furthermore, the positions and intensities of these peaks align well with reported values in the literature, reinforcing the accuracy and reliability of the synthesis process. The combination of MWCNTs and ZnO in the nanofibers results in a composite material with enhanced structural and functional properties, as confirmed by this detailed XRD analysis [71,72]. This structural characterization highlights the potential of the MWCNT-ZnO nanofibers for advanced applications that require both high crystallinity and the synergistic benefits of these two materials.

FTIR spectroscopy was used to analyze the functional groups in the MWCNT-ZnO composite, providing crucial insights into the chemical bonds within the material. The spectrum, shown in Figure 3D, was measured over a range of 500 to 4000 cm^−1^, revealing distinct peaks that characterize the molecular interactions. The peak at 662 cm^−1^ represents Zn-O bending vibrations, confirming the presence of zinc oxide within the nanofibers. The broad peak at 3366 cm^−1^ is attributable to Zn-OH stretching vibrations, indicating the existence of zinc hydroxide groups. The peak at 1107 cm^−1^ corresponds to the O-C stretching bond, demonstrating the involvement of oxygen-carbon groups in the composite. The carbon-based nature of the MWCNTs is evidenced by the peak at 1497 cm^−1^, which represents C=C bending vibrations. Another peak, found at 1390.23 cm^−1^, is linked to C-O-H bending, signifying the presence of hydroxyl groups. A peak at 2104 cm^−1^ is associated with -C-C- stretching vibrations, typical of carbon–carbon bonds found in nanotubes. The high wavenumber peak at 3745 cm^−1^ is attributable to hydroxyl (OH) groups, further indicating the presence of carboxyl functionalities, which are important for the composite’s potential interactions. Slight shifts observed in these peaks suggest significant interactions between the MWCNTs and ZnO, possibly due to doping. This FTIR analysis highlights the successful incorporation of both ZnO and MWCNT components within the composite, verifying the presence of key functional groups [32,73]. This integration of functional groups increases the composite’s chemical reactivity and enhances its suitability for advanced applications, such as sensor technology and catalysis, where surface interaction plays a critical role.

### 3.2. Characterization of CuO Micro-Flowers

**Figure 4:**
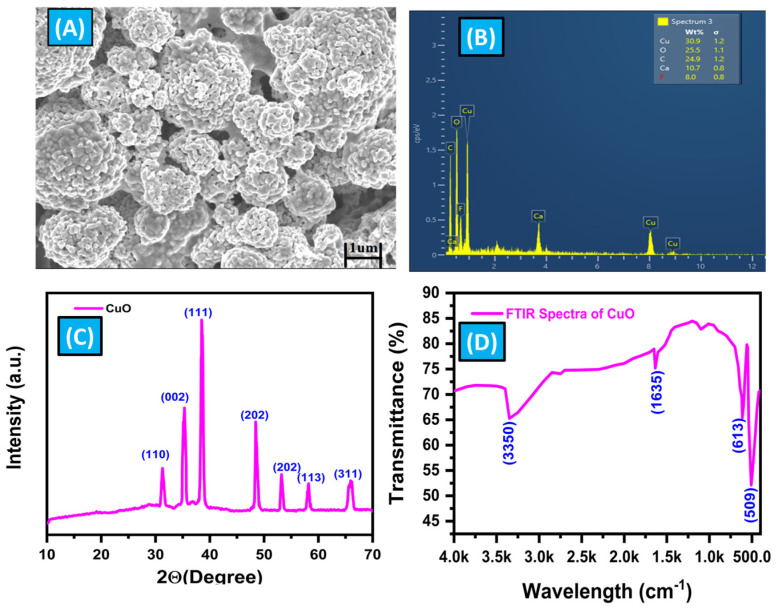
(**A**) 3D pictures of the CuO-MFs, derived from a morphological study performed using scanning electron microscopy (SEM). (**B**) EDX spectra of CuO-MFs. (**C**) XRD spectra of CuO-MFs. (**D**) FTIR spectrum of CuO-MFs.

The characterization of CuO-MFs was conducted using SEM, XRD, EDX, and FTIR, as illustrated in Figure 4. The SEM image, as shown in Figure 4A, reveals that the CuO-MFs consist of numerous flower-like aggregates made up of small irregularly shaped particles, with diameters ranging from approximately 1 to 10 µm. Each microflower is composed of many smaller agglomerated particles, with sizes around 100 nm, as confirmed by XRD analysis. Figure 4B shows the EDX spectra of CuO-MFs. The EDS analysis showed a 1:1 atomic ratio of copper (Cu) to oxygen (O), indicating that the synthesized CuO-MFs have a pure composition. However, we also detected calcium (Ca) and fluorine (F) in the results. These elements might be contaminants from the sample preparation process. Even though we used double distilled water to make the solution, some impurities may have come from washing or handling the sample slide preparation process. Therefore, it is found in EDX spectra. The XRD pattern presented in Figure 4C confirms the crystalline structure of the synthesized CuO nanoparticles. Sharp peaks in the XRD plot, observed at 2θ values between 30.5° and 67.5°, correspond to the crystal planes (111), (002), (120), (202), (122), and (222). Notably, reflections at 2θ = 30.896° (111) and 2θ = 35.8265° (002) further confirm the formation of the monoclinic crystal phase of CuO. The calculated crystallite size is approximately 20 nm, indicating successful synthesis of the nanoparticles with a well-defined crystalline structure. The results obtained from this characterization align well with the previously reported literature [63,64,65].

The FTIR spectrum shown in Figure 4D demonstrates the distinctive vibrations of CuO nanoparticles produced via a sol-gel technique utilizing CuSO_4_ as a precursor, followed by calcination at 500 °C. The spectrum was acquired by sequentially measuring wavelengths ranging from 4000 to 400 cm^−1^. Within this spectrum, there are two distinct peaks observed between 509 and 613 cm^−1^, which can be attributable to the stretching vibrations of the CuO bonds in a monoclinic crystal structure. In addition, there are less intense bands seen in the range of 1700 to 1300 cm^−1^, which can be attributed to the symmetrical stretching vibrations of Cu-O bonds in the nanoparticles. The FTIR spectra show a clear absorption peak at 3350 cm^−1^, which is caused by the O-H stretching vibrations of water molecules that are adsorbed onto the surface of the CuO-MFs. The obtained FTIR characterization results were well-matched with earlier reported literature studies [68,69,74]. The discoveries are vital for comprehending the chemical production procedure and the structural attributes of CuO-MFs, which are pivotal for their utilization in diverse domains such as catalysis, sensing, and energy storage.

### 3.3. Morphology and Structural Studies of Functionalized Sensor

**Figure 5:**
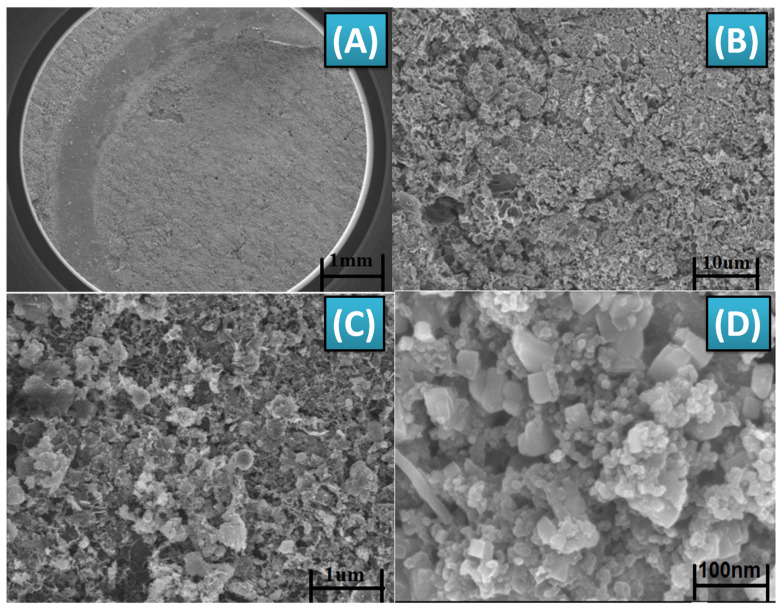
SEM image of MWCNT-ZnO/CuO micro-flower deposition on working electrode of Integrated Graphene IG-GII-SENS-01 SPE at magnification levels of (**A**) 1 mm, (**B**) 10 µm, (**C**) 1 µm, and (**D**) 100 nm.

The FESEM image in Figure 5 illustrates the Gii-Sens Integrated Graphene screen-printed working electrode, which has been functionalized with MWCNT-ZnO and well-dispersed CuO-MFs in a Nafion solution. These SEM images provide valuable information about the size, morphology, and uniformity of particle dispersion on the sensor substrate. Figure 5A shows the arrangement of MWCNT-ZnO nanofibers and CuO-MFs, highlighting their distribution, clustering, and presence on the electrode surface. Figure 5A shows the low-magnification image reveals that the entire working electrode area is thoroughly coated with CuO-MFs and MWCNT-ZnO nanofibers, which exhibit consistent and uniform shapes. The surface morphology depicted in Figure 5B appears porous, contributing to the sensor’s performance. Figure 5C presents a highly magnified FESEM image of the CuO-MFs, providing further details about their structure. Figure 5D shows the uniform distribution of MWCNT-ZnO buried below the CuO-MFs. This combination of materials enhances the overall functionality of the biosensor.

### 3.4. Electrochemical Cyclic Voltammetry Characterization of the MWCNT-ZnO/CuO-MFs Modified SPEs

**Figure 6:**
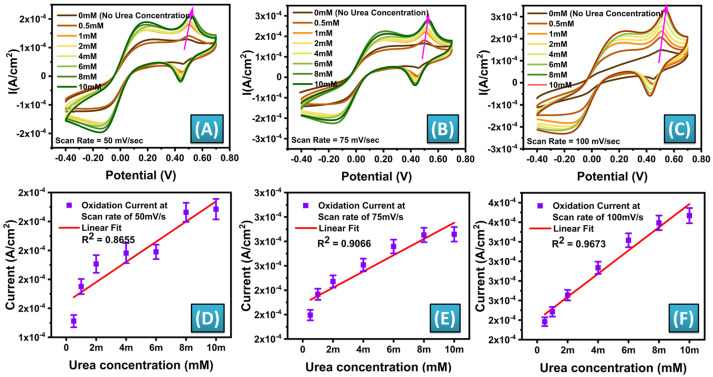
Cyclic voltammetry (CV) responses of the MWCNT−ZnO/CuO−MFs modified SPEs were measured at various concentrations of urea ranging from 0.5 to 10 mM in the presence of a 5 mM solution of Ferroferricyanide [Fe(CN)_6_]^3−4−^ as the standard redox probe at scan rates of (**A**) 50 mV/s, (**B**) 75 mV/s, and (**C**) 100 mV/s. Corresponding calibration plot of urea concentration (mM) versus current (A/cm^2^) for a scan rate of (**D**) 50 mV/s, (**E**) 75 mV/s, (**F**) 100 mV/s (n = 5).

The electrochemical behavior of MWCNT-ZnO/CuO-MF nanocomposite-modified screen-printed electrodes (SPEs) was investigated using cyclic voltammetry (CV) with a solution containing 5.0 mM ferro/ferricyanide [Fe(CN)_6_]^3−/4−^, a typical redox probe. The electrodes that were enhanced with MWCNT-ZnO/CuO-MFs had unique amperometric characteristics, as illustrated in Figure 6A–C. The experiments involved the analysis of urea samples using cyclic voltammetry at different concentrations, ranging from 0.5 to 10 mM. The CV curves were plotted with scan rates of 50, 75, and 100 mV/s, covering a potential range from −0.4 to + 0.7 V. Figure 6 depicts the results of these experiments under various circumstances.

Electrochemical CV is a powerful method used to investigate electrochemical reactions occurring on electrode surfaces. The process entails systematically varying the applied voltage to the electrode in a cyclic manner while simultaneously measuring the resulting electric current. The morphology and attributes of the CV curves offer valuable insights into the electrochemical activities taking place at the electrode interface. The scan rate affects the maximum currents measured in the CV curves, which indicate the speed of the redox processes and the movement of species in the solution. The addition of MWCNT-ZnO/CuO-MF nanocomposites to the SPEs improves the electrochemical performance, likely due to the increased surface area and enhanced electron transfer kinetics. This modification enhances the ability to detect and analyze urea concentrations across a broad range accurately. It showcases the adaptability and efficiency of the electrodes that have been modified with nanocomposites in electrochemical sensing applications.

The calibration curve for the redox current was plotted at scan rates of 50, 75, and 100 mV/s, as seen in Figure 6D–F. The calibration curve revealed that the highest regression coefficient (R^2^) was achieved at a scan rate of 100 mV/s. The maximum R^2^ value at the scan rate of 100 mV/s indicates the unique peak current value obtained for an individual urea concentration. At a scan rate of 100 mV/s, a linear calibration curve was achieved for the concentration range of 0.5–8 mM. The sensitivity of the SPE biosensor modified with MWCNT-ZnO/CuO-MFs composite is calculated using Equation (1) [75]:

(1) $$Sensitivity=\frac{Slope\;of\;Calibration\;plot}{Active\;Surface\;Area\;of\;the\;Sensor}$$

The sensor, functionalized with an MWCNT-ZnO/CuO-MF nanocomposite on a working electrode with a sensing area of 0.1257 cm^2^, exhibited a sensitivity of 117.98 mA mM^−1^ cm^−2^ for the non-enzymatic detection of urea.

### 3.5. Selectivity and Stability Study of the Sensor

**Figure 7:**
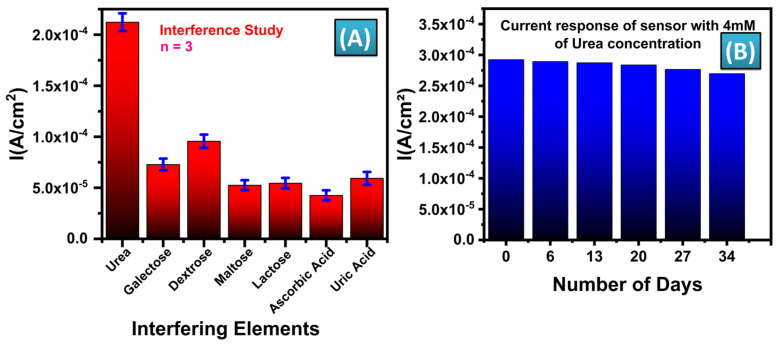
(**A**) Selectivity study with commonly identified interfering elements in human blood such as Galactose, Dextrose, Maltose, Lactose, Ascorbic Acid, and Uric Acid with a concentration of 0.1 mM at the scan rate of 100 mV/se, (**B**) Stability study of MWCNT−ZnO/CuO−MFs modified sensor for urea detection.

A selectivity study was conducted to assess the interference of common blood elements—Galactose, Dextrose, Maltose, Lactose, Ascorbic Acid, and Uric Acid—each at a concentration of 0.1 mM, with respect to urea detection at a concentration of 1 mM. Given that the normal concentrations of these interfering elements in human blood are significantly lower than the typical urea concentration (ranging from 1.67 to 7.5 mM), this study aimed to evaluate the sensor’s specificity for urea. CV was performed on separate sensors spiked individually with the mentioned interferents, and the corresponding current peaks were measured. Figure 7A illustrates the current response at 0.5 V for these interfering elements, compared with urea at a 1 mM concentration, all tested at a scan rate of 100 mV/s. In addition, it was observed during the experimentation that all the interferents tested show the maximum oxidation current at the potential of nearly 0.18 V, whereas, in our case the urea oxidation takes place at 0.5 V. The results confirm that the sensor maintains selectivity towards urea despite the presence of potential interferents. The interference tests were conducted within a potential window of −0.4 V to 0.7 V, and the findings are presented in Figure 7A, demonstrating that the device’s response to urea remains robust, validating its selectivity in complex biological matrices.

The durability of the MWCNT-ZnO/CuO-MF functionalized SPE biosensor modified for urea detection was assessed by conducting CV measurements for a period of 34 days. The experiments were performed using 4 mM urea concentration. Figure 7B demonstrates that the current magnitude, when subjected to a scan rate of 100 mV/s, shows 1.06% and 1.70% of its initial values after seven and fourteen days, respectively. Similarly, the percentage change in current magnitude observed after the 20th, 27th, and 34th days was 3.07%, 5.46%, and 7.75%, respectively. The results suggest that the MWCNT-ZnO/CuO-MF functionalized urea sensor has good storage stability. The improved biocompatibility of MWCNT-ZnO/CuO-MF functionalized SPEs facilitates the establishment of a stable environment, enabling the sensor to maintain its bioactivity for an extended period. The stability of the biosensor is essential for the practical use of long-term urea detection, guaranteeing consistent and dependable performance during storage.

### 3.6. Machine Learning Approach for Prediction of Urea Concentration

ML techniques can assist in calibrating biosensors by learning from calibration data sets and adjusting sensor responses accordingly. Moreover, ML can integrate data from multiple sensors (sensor fusion), enhancing accuracy and reliability by combining complementary information. ML algorithms can enable real-time monitoring of analyte concentrations or biological parameters, providing continuous feedback. Predictive models can anticipate changes based on historical data, facilitating proactive interventions in medical or environmental applications. This study and analysis of various ML models in this scenario aims to enhance predictive performance beyond what traditional linear regression (LR) offers, particularly in the realm of sensor data analysis.

**Figure 8:**
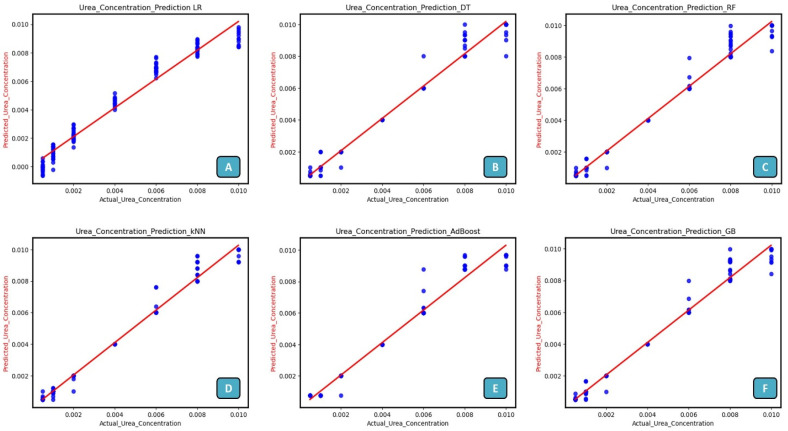
Prediction of urea concentration using machine learning algorithms: The available machine learning models are (**A**) LR, (**B**) DT, (**C**) RF, (**D**) KNN, (**E**) AdaBoost, and (**F**) GB.

The urea detection with the ML approach was successfully used to improve the sensor’s accuracy and predict the urea concentration. These data, resulting from more than 200 experiments, were utilized to train and evaluate the ML models. The ML model was trained on eighty percent of the data sets; the other twenty percent were new to the model and were kept for testing. The ML models were trained (considering current as an input) using data gathered from different sensors, with an emphasis on data falling into the linear range. As shown in Figure 8, regression-based analysis was carried out, and the performance of various ML models, including LR, decision tree (DT), random forest (RF), K-nearest neighbor (KNN), gradient boost (GB), adaptive boosting (AdBoost) models were compared using various regression metrics such as mean absolute error (MAE), mean squared error (MSE), root mean squared error (RMSE), and coefficient of determination (R-squared). The regression metrics are determined by the following equations [63,64].

(2) $$Mean\;Absolute\;Error=\frac{\sum_{i=1}^{N}\left|Y_{i}^{Pred}-Y_{i}^{act}\right|}{N}\ldots ..$$

(3) $$Mean\;Squared\;Error=\frac{1}{N}\sum_{i=1}^{N}(Y_{i}^{pred}-Y_{i}^{act}{)}^{2}\ldots \ldots .\;\;\;\;\;\;\;\;\;$$

(4) $$Root\;Mean\;Squared\;Error=\sqrt{\frac{\sum_{i=1}^{N}(Y_{i}^{pred}-Y_{i}^{act}{)}^{2}}{N}}\ldots \ldots .$$

(5) $$Coefficient\;of\;Determination=1-\frac{\sum_{i=1}^{N}(Y_{i}^{pred}-Y_{i}^{act}{)}^{2}}{\sum_{i=1}^{N}(Y_{i}^{pred}-Y^{\prime }{)}^{2}}\ldots \ldots$$

The performance metrics for different regression-based ML models are tabulated in Table 2. ML models such as LR, DT, RF, KNN, AdaBoost, and GB outperform LR in predictive accuracy metrics such as MSE, RMSE, and R^2^ Score. For instance, models such as KNN exhibit significantly lower MSE (1.93) and RMSE (0.0004) compared with LR (MSE 4.81, RMSE 0.0006), indicating they produce more precise predictions. Sensors often capture data with complex, non-linear relationships that LR may struggle to capture effectively. DT and ensemble methods such as RF and GB excel in capturing such complexities by dividing data into hierarchical structures or aggregating multiple weak learners to form robust predictions. ML models generally exhibit better robustness to outliers and noisy data compared with LR, which can be sensitive to such deviations. This robustness is reflected in lower MAE values across most ML models compared with LR. The R^2^ Score measures how well the model explains the variance in these data. ML models consistently achieve higher R^2^ Scores than LR (e.g., KNN and RF both at 0.979), suggesting they provide a better fit to these data and capture more variance. Based on the provided metrics, models such as KNN, RF, and GB consistently demonstrate superior performance in terms of accuracy and explanatory power (R^2^ Score) compared with LR. This indicates that for sensor data where accuracy and understanding of variance are crucial, these ML models can provide more reliable and insightful predictions.

## 4. Conclusions

This study successfully demonstrates the development of a flexible, non-enzymatic electrochemical biosensor for urea detection, incorporating ML for enhanced accuracy. The sensor utilizes an MWCNT-ZnO nanocomposite functionalized with CuO-MFs to modify screen-printed electrodes. Through electrochemical characterization using electrochemical CV, the sensor exhibits excellent performance with a linear detection range of 0.5–8 mM and a low limit of detection (LoD) of 78.479 nM, making it highly sensitive (117.98 mA mM^−1^ cm^−2^) for detecting urea concentrations within physiological levels. The incorporation of ML models, including random forest, K-nearest neighbor (KNN), gradient boosting, and adaptive boosting, significantly enhanced the accuracy of urea concentration predictions. Data from over 200 experiments were used to train and evaluate the models, with regression metrics such as mean absolute error (MAE) and root mean squared error (RMSE) validating the model performance. The integration of ML not only improved sensor accuracy but also enabled predictive analysis, optimizing the sensor’s response to different urea concentrations. Overall, this work presents a highly sensitive and reliable biosensing platform that leverages the combined advantages of nanomaterial-based sensor design and machine learning. This approach offers promising applications in real-time monitoring and point-of-care diagnostics for kidney function and other urea-related health conditions, paving the way for future advancements in biochemical sensing technologies.

## Figures and Tables

**Table 1:** Comparison of MWCNT-ZnO/CuO-MFs modified non-enzymatic biosensors with earlier reported biosensors for urea detection.

Electrode Material Used for Urea Detection	Machine Learning	Sensitivity	Limit of Detection	Linear Range	Reference
Ag/ZnO nanorod	No	0.1622 μAμM^−1^ cm^−2^	13.98 μM	26.3 to 427 μM	[57]
Gr-PANi	No	−226.9 μA/μM cm^2^	5.88 μM	10 μM–200 μM	[49]
Ni-MOF/MWCNT	No	685 μAmM^−1^ cm^−2^	3 μM	0.01–1.12 mM	[58]
Ag-N-SWCNTs	No	141 μAmM^−1^ cm^−2^	4.7 nM	66 nM to 20.6 mM	[53]
LaNi_0.6_Fe_0.4_O_3_-CeO_2_ (LNF-C)/MWCNT/ITO	No	195.6 μAmM^−1^ cm^−2^	1 μM	25 to 670 μM	[59]
NiO Nanosheets		3.4 A/M cm^2^	2 μM	4.4 μM to 181.6 μM	[60]
ZnO@rGO	No	682.8 μA mM^−1^ cm^−2^	0.012 μM	0.02 × 10^−3^ mM to 7.2 × 10^−3^ mM	[61]
CuO/Co_3_O_4_@ MWCNTs	No	--	0.223 pM	10^−12^ to 10^−2^ M	[24]
CuO/c-MWCNT/GCE	No	23.8983 µA/mM	0.16 mM/L	2 mM^–8^ mM	[62]
MWCNT-ZnO/CuO-MFs	Yes	117.98 mA mM^−1^ cm^−2^	78.479 nM	0.5 mM to 8 mM	This Work

Ag: Silver, Gr: Graphene, PANi: Polyaniline, Ni-MOF: Nickel-metal organic framework, SWCNTs: Singled walled Carbon Nanotubes, LaNi_0.6_ Fe_0.4_O_3_-CeO_2_, LNF-C: perovskite-type oxide, ITO: Indium Tin Oxide, rGO: Reduced Graphene Oxide, Co_3_O_4_: Cobalt oxide, GCE: Glossy Carbon Electrodes.

**Table 2:** Urea concentration prediction using ML algorithms.

ML Models	Various ML Model Regression Accuracy Parameters			
	MAE	MSE	RMSE	R^2^ Score
LR	0.0005	4.81	0.0006	0.953
DT	0.0001	2.20	0.0004	0.978
RF	0.0001	2.12	0.0004	0.979
KNN	0.0001	1.93	0.0004	0.981
AdaBoost	0.0003	2.84	0.0005	0.972
GB	0.0001	2.13	0.0004	0.979

## Data Availability

The original contributions presented in this study are included in the article; further inquiries can be directed to the corresponding authors.
